# Parenting Influences on the Development of Alcohol Abuse and Dependence

**Published:** 1997

**Authors:** Theodore Jacob, Sheri Johnson

**Affiliations:** Theodore Jacob, Ph.D., is a career research scientist at the Palo Alto Veterans Administration Health Care System, Palo Alto, California. Sheri Johnson, Ph.D., is an assistant professor of psychology at the University of Miami, Coral Gables, Florida

**Keywords:** children of alcoholics, family environment, parenting skills, AODU (alcohol and other drug use) development, AOD use behavior, offspring, role model, expectancy, parent child relations, antisocial behavior, conduct disorder, emotional and psychiatric depression, alcohol use disorder classification, literature review

## Abstract

Both alcohol-specific and non-alcohol-specific parenting influences affect the development of alcohol abuse and dependence in the offspring. Alcohol-specific influences (e.g., the modeling of parental drinking behavior, the development of alcohol expectancies, and certain aspects of the parent-child relationship) are particularly relevant to the development of alcohol abuse and dependence in children of alcoholics. In contrast, non-alcohol-specific influences generally promote deviant behavior, including alcohol problems, in the offspring and affect children of alcoholics and nonalcoholics equally. These influences, which include inadequate parenting and other parent-child interaction patterns that promote aggressive, antisocial behavior in children, increase the offspring’s risk of an alcoholism subtype associated with antisocial personality disorder. A different set of non-alcohol-specific family influences may contribute to an alcoholism subtype that emerges after the onset of depression.

Over the past century, researchers increasingly have explored the family’s role in the development (i.e., etiology), course, treatment, and prevention of alcohol abuse and dependence (Windle and Searles 1990). Much of this research has focused on the children of alcoholics (COA’s), who are at a significantly higher risk of becoming alcoholic themselves than are the children of nonalcoholics (non-COA’s). Various perspectives have been used to guide empirical studies in this area, including genetic-biological efforts; studies of the personality and psychosocial characteristics of the alcoholic as well as of his or her spouse and children; and analyses of other variables (i.e., moderator variables), such as family structure, social class, and ethnicity.

This article focuses on influences originating in the family environment—particularly parenting influences—that are relevant to the development of alcohol abuse and dependence in the offspring.^1^ This discussion includes two types of family influences: alcohol-specific and non-alcohol-specific effects. Alcohol-specific effects reflect the impact of parental alcohol use and abuse on the child’s alcohol use and abuse behavior; accordingly, these influences are more relevant to COA’s than to non-COA’s. Non-alcohol-specific effects, in contrast, include more general features of the family environment that increase the child’s risk for deviant behavior, including alcohol abuse. As a result, these influences affect all children and adolescents and are not specific to COA’s. Some non-alcohol-specific influences, however, also may have a particularly strong impact on COA outcome, because certain factors contributing to these effects (e.g., parental psychopathology) are more prevalent among COA’s than among non-COA’s. The strongest model of the role of family environmental influences in the development of alcoholism emphasizes these non-alcohol-specific effects.

## Alcohol-Specific Influences

Numerous studies have demonstrated that the drinking patterns of parents and their adolescent and adult children are highly correlated. For example, one study found that adolescent COA’s are 5.1 times more likely than non-COA’s to report a social consequence or dependence symptom related to alcohol and other drug (AOD) use (Chassin et al. 1991). Considerable uncertainty still exists, however, regarding *how* parental alcohol abuse increases the children’s risk for alcohol problems. Research to determine the influence of parental drinking has evaluated individual, dyadic, and family-level effects.

Individual effects represent the impact of one person’s behavior on the child’s outcome. Studies of these effects have focused on the roles of parent modeling and alcohol expectancies in shaping COAs’ drinking behavior. These analyses address questions such as the following: Do children model their parents’ drinking behavior? What alcohol expectancies do COA’s have (i.e., how do they expect to feel when they drink alcohol)? (For more information on these issues, also see the article by Ellis et al., pp. 218–226.) Dyadic effects result from the interactions of two family members. Studies of dyadic effects have focused on the parents’ marital relationship, the parent-child relationship, and the child’s relationship with his or her siblings. A few lines of research also have highlighted family level effects, such as the overall family climate and interactional patterns.

### Individual Effects

Although extensive developmental literature exists describing children’s modeling of various parental behaviors, relatively little research has investigated this topic among COA’s. Nonetheless, findings of a strong link between the quantity of alcohol use in children and their parents suggest that this area warrants further attention. More research also is necessary to understand whether different patterns of parental drinking have implications for drinking style and problems among COA’s. For example, are children of binge-drinking alcoholics more likely to exhibit that same drinking style? Previous studies have suggested that the extent to which a child models the behavior of his or her parent(s) also is moderated by the warmth and degree of reinforcement shown by the parent(s) (Bandura 1969). Accordingly, alcohol researchers must consider not only drinking patterns but also family relationships when evaluating the role of modeling in determining COAs’ drinking behavior.

Recent studies have suggested that a person’s beliefs regarding the effects of alcohol (i.e., cognitive alcohol expectancies) can be important predictors of alcohol use and abuse. Furthermore, studies assessing the impact of parental drinking on the development of children’s alcohol expectancies have revealed the following:

- Children’s perceptions of parental drinking quantity and circumstances appear to influence their own drinking frequency (Brook et al. 1990; Kandel and Andrews 1987).
- Children’s alcohol expectancies reflect recognition of alcohol-related norms and a cognizance of parental drinking patterns by a very early age (Zucker et al. 1995).
- Alcohol expectancies appear to be one of the mechanisms explaining the relationship between paternal alcoholism and heavy drinking among offspring during college (Sher et al. 1996).
- Changes in parental drinking patterns result in changes in the nature and impact of children’s alcohol-related expectancies. In a 3-year study of adolescent COA’s, those whose fathers no longer experienced alcohol-related problems demonstrated a stronger relation between their beliefs in restrained drinking and lowered alcohol use than did COA’s whose fathers continued to experience alcohol-related problems. Moreover, belief in drinking restraint predicted lower AOD consumption (Chassin and Barrera 1993).

These findings suggest that the role of expectancies in shaping drinking patterns should be explored further. Such studies also should address how family environments may moderate the relationship between the parent’s drinking style and the child’s cognitive expectancies.

### Dyadic Effects

Of the various family dyads, the parent-child relationship has received the most attention in the study of alcohol-specific family influences, although the role of sibling relationships also has been evaluated (see box, p. 206). Several studies have assessed the quality of the parent-child relationship in families with an alcoholic parent to learn more about the factors that contribute to heavy drinking by adolescent COA’s (Chassin et al. 1993, 1996; Kandel and Andrews 1987). The results of these investigations suggest that paternal alcohol abuse may be associated with decreased monitoring of the children’s behavior. This particular facet of parenting, in turn, is a risk factor for increased association with AOD-using peers, and thereby for AOD use, among the children. These findings indicate that further clarification of parent-child relationships will enhance our understanding of the intergenerational transmission of alcoholism.

**The Role of Siblings in Shaping Alcohol and Other Drug Use Patterns**

Studies indicate that siblings are moderately alike in their alcohol and other drug (AOD) use patterns. Brook and colleagues (1990) describe three potential pathways through which siblings’ AOD use patterns could be related. First, an older sibling may influence a younger sibling through modeling processes (i.e., the younger child models the older child’s behavior). As a result, the siblings would share similar attitudes, values, and behaviors that could lead to similarities in AOD use. Second, siblings share approximately 50 percent of their genetic material. Consequently, they may have inherited the same genetic predisposition for AOD use and thereby experience similar outcomes. Third, a positive, healthy relationship with an older sibling may result in less conflict and distress for a younger sibling, thereby promoting more conventional attitudes and behavior and subsequently reducing the risk of AOD use. In addition to these mechanisms, Rowe and Gulley (1992) have hypothesized that an older sibling’s choice of friends significantly influences the younger child’s social environment, thus influencing the child’s degree of deviant behavior and drinking patterns. Finally, factors in the shared family environment, such as the quality of parenting, likely influence all siblings in the same way, leading to similar outcomes. More studies are needed, however, to confirm these findings and to further clarify the forces associated with these similarities in sibling AOD use patterns.

### Family-Level Effects

To date, relatively little research has evaluated family characteristics, such as communication, warmth, and cohesion, that may influence the risk of drinking problems in COA’s. In a notable exception, Wolin and colleagues (1980) have suggested that the extent to which drinking disrupts family rituals, such as holiday events and daily routines, predicts the children’s risk for alcohol abuse. This line of research shows the value of tracing differences among alcoholic families as a way of better understanding why some, but not all, COA’s develop AOD use-related difficulties.

Family interaction patterns also may influence the COA’s risk for alcohol abuse. For example, Jacob and Krahn (1988) found that families with an alcoholic parent displayed more negative family interaction during problem-solving discussions than did control families in which the parents exhibited no alcoholism or major psychopathology. In addition, parental binge drinking appears to be associated with more disturbed family interactions than does steady drinking (Jacob and Leonard 1988). These observations indicate the importance of assessing a broad range of family and social characteristics to facilitate the prediction of which COA’s are most at risk for future alcohol problems (Dobkin et al. 1997).

## Non-Alcohol-Specific Influences

In addition to parental drinking, a broader range of family influences is associated with both alcohol problems and externalizing behaviors (e.g., antisocial behavior and aggression). For example, the family backgrounds of AOD abusers frequently are characterized by marital instability, lack of support, poor discipline, and family conflict. Although these family problems are common in alcoholic families and therefore may contribute significantly to the COAs’ risk for AOD problems, they also can affect the outcomes of non-COA’s. Each of these areas of family difficulty has received empirical attention; however, numerous studies have suggested that difficulties in the parent-child relationship may be the most important pathway through which various family factors influence the child’s outcome (Fauber et al. 1990). Accordingly, the following sections focus on parenting as the aspect of family functioning most significantly associated with AOD abuse both in COA’s and non-COA’s.

### General Parenting Effects on Child Outcome

The primacy of the family in regard to the child’s social and cognitive development has been the cornerstone of the child development and family studies literature for more than 50 years. The distillation of this voluminous literature yields two particularly relevant conclusions. First, all familial variables that can affect child outcome—for example, parental dispositions, marital and sibling influences, and the sociocultural context in which the family operates—are played out within the interactions between the parent and the child. Second, the parent-child interaction is characterized primarily by two major parenting dimensions: nurturance (i.e., warmth and support) and control (i.e., supervision and discipline). Disturbances in either or both of these parenting dimensions can have severe and wide-ranging effects on the child’s social-emotional and cognitive development.

Most important, inadequate parenting, which is characterized by lack of affection and/or high levels of criticism and hostility, lax or inconsistent discipline and supervision, and general lack of involvement, provides the foundation for the development of an aggressive, antisocial behavior pattern. As early as the preschool years, such a pattern can manifest itself in the form of noncompliance. Over time, and with ongoing parenting difficulties, noncompliance evolves into a behavior pattern characterized by early peer rejection, poor academic performance, delinquency, AOD abuse, and association with deviant peers. Over the years, the child socialization model just described has received a great deal of support, particularly from studies on the development of adolescent AOD abuse and delinquency. Although numerous factors contribute to the etiology of alcoholism, studies of this model have focused mainly on examining the behavioral interactions between the parent and child. These analyses have led to the continued refinement of the psychosocial theory, which links family process with child outcome.

### Parenting Effects on Alcohol Abuse of Offspring

Strong associations exist between child conduct disorder, adolescent delinquency, adult antisocial behavior, and alcohol abuse, as well as between adult antisocial behavior and adult alcoholism (see Jacob and Leonard 1994). For example, almost 20 percent of alcoholics meet the criteria for antisocial personality disorder (ASPD), which is characterized by a pervasive pattern of disregard for and violation of the rights of others. In fact, ASPD-associated alcoholism is the most common alcoholism subtype. People with this pernicious form of alcoholism display more severe social, familial, and occupational impairments than those without comorbid ASPD. Moreover, ASPD is 21 times more common in people who abuse or are dependent on alcohol than in people without alcohol abuse or dependence (Zucker 1994). Accordingly, the factors contributing to the etiology of ASPD-associated alcoholism have been a focus of intense research.

The close association between ASPD and alcoholism suggests that parent-child interactions that promote aggressive, antisocial behavior (e.g., inadequate parenting) play a significant role in the alcoholism etiology of both COA’s and non-COA’s. Developmental research consistently has suggested that ASPD-associated alcoholism emerges after various antisocial behaviors have already been established (Zucker 1994). Consequently, this alcoholism subtype can be viewed best within the context of understanding the development of the broader syndrome of antisocial behavior. In fact, many researchers consider family variables that predict the development of a generalized deviant-behavior syndrome the most powerful indicators of the development of alcohol abuse and alcoholism.

Various models have been established to explain the relationship between parenting influences and childhood conduct disorders. The most influential model, which was developed by Patterson and colleagues (1992), posits that among the numerous forces determining a child’s social and emotional development, disturbances in the control dimension are most relevant to the development of undercontrolled behavior. This model emphasizes particularly the impact of a coercive interactional style between parent and child. A coercive interactional style is marked by inconsistent reinforcement for good behavior and unclear behavioral expectations from the parents and lack of compliance by the children. Continuation of this coercive relational style results in further parental rejection and less contact with the child throughout the preadolescent years, as well as in inadequate parental monitoring, discipline, supervision, and communication skills during the child’s adolescence. During late childhood and adolescence, these family patterns can lead to antisocial behavior, which, in turn, increases the likelihood of AOD abuse. Slowly, but steadily, this coercive interactional style also extends to the child’s relationships with peers and teachers, increasing the probability of peer rejection, academic difficulty, and association with deviant (i.e., antisocial) peer groups. This broadening scope of problem behavior only intensifies the coercive, negative parent-child interactions.

Empirical support for the hypothesized relationship between inadequate parenting and the development of aggressive, antisocial behavior in childhood and adolescence comes from studies of child psychopathology and its treatment. These analyses have documented specific parent-child interactions that precede and are associated with the emergence of aggressive, antisocial behavior (Patterson et al. 1992; Snyder and Huntley 1990; Wahler and Dumas 1987). In addition, long-term (i.e., longitudinal) investigations of delinquency and its development have repeatedly identified inadequate parenting in the early childhood histories of people who subsequently develop antisocial behavior and alcohol abuse during adolescence and adulthood (Zucker 1994).

Another longitudinal study of adolescents also has focused on parenting variables as the major psychosocial influence in the child’s development of AOD use and abuse patterns (Brook et al. 1990). Several findings of this study further elucidate the association between parenting style and adolescent AOD use, as follows:

- The level of mutual warmth, support, and control within the parent-adolescent relationship significantly predicted the risk of adolescent drug (i.e., marijuana) use.
- Adolescent personality characteristics such as sensation seeking, rebelliousness, and tolerance for deviance were robust predictors of adolescent AOD use.
- A positive relationship (i.e., attachment) between the parent and adolescent served as a protective factor, offsetting the risk of AOD use associated with peer AOD use.

These findings further support the notion that parental warmth and monitoring are critical components in predicting the child’s risk of AOD use (also see Kandel and Andrews 1987).

### Parenting Influences Relevant to Other Alcoholism Subtypes

Just as the well-developed literature on the relationship between family influences and aggressive, antisocial behavior informs about one alcoholism subtype, literature relevant to other problem behaviors and psychopathologies may inform about other alcoholism subtypes that may be predicted by different family characteristics. One of these alcoholism subtypes is called negative-affect alcoholism. This type of alcoholism, which occurs more commonly in women than in men, is characterized by the emergence of alcohol problems after the onset of depression and/or anxiety disorders (Hesselbrock et al. 1986), suggesting that depression may increase the risk of alcoholism. This hypothesis is further supported by findings that alcoholics are twice as likely as the general population to have an anxiety or affective disorder (King et al. 1991).

Research on depression has led to the development of several family models and the identification of family characteristics associated with depression, which may help elucidate the etiology of negative-affect alcoholism. These studies found that family influences contribute significantly to the development of depression. For example, parental depression is a strong predictor of offspring depression (Hammen et al. 1990). Depression among the parents also may lead to less positive patterns of family interaction (Jacob and Johnson in press). Finally, parents with depression may model maladaptive coping and cognitive styles. Thus, depressed parents may be particularly likely to exhibit coping styles that deemphasize problem-solving as well as to express negative beliefs regarding themselves, their future, and their world. Children who are exposed to these cognitive and coping styles may internalize them, thereby increasing their own risk for depression. As these models of parental influences on the child’s risk for depression are replicated and elaborated, it will be important to integrate this information into ongoing research on negative-affect alcoholism.

## Conclusions

It is now well established that the family environment, and particularly parenting effects, strongly influence a child’s risk of alcohol abuse and dependence. The most comprehensive and substantial evidence for such effects comes from social learning theory-based research on the development of aggressive, antisocial behavior and from longitudinal research that has traced family and peer influences on the development of AOD abuse. The findings of these analyses have led to a reasonably clear consensus that family environment and children’s alcohol use are intertwined, especially in families with an alcoholic parent.

Although COA’s generally are at increased risk of becoming alcoholic themselves, more than one-half of COA’s exhibit no alcohol problems, and not all families with an alcoholic parent exhibit impaired parenting behavior. Furthermore, although certain parenting styles may increase COAs’ risk for deviant behavior, including AOD use, many COA’s show great resilience in these difficult environments and either develop no behavior problems or mature out of them during early adulthood. In short, great heterogeneity and variability exist among COA’s and their families as well as among their outcomes.

To more accurately predict which COA’s are most at risk for alcohol problems, researchers must improve their understanding of the mechanisms that translate the family environment into this risk. Some promising lines of research have examined the influences exerted by family interaction and disturbances of daily and holiday routines. However, the resulting models of the pathways linking parental alcohol abuse with child outcome still must be replicated and extended. In addition, researchers need to examine moderating factors that may increase or decrease COAs’ risk within the context of parental alcoholism. Factors that appear to be particularly important potential moderators include parent-child interactions, parental personality, comorbid parental symptomatology, and child personality and temperament.

These family factors must be assessed in the context of other developmental influences on the children that originate outside of the family. Complex links exist between parent-child relationships, child personality traits, and peer relationships that influence the onset and continuance of AOD use. For example, the parent-child relationship and parental alcohol abuse influence the type of friends an adolescent chooses (Kandel and Andrews 1987). Peer relationships, in turn, predict AOD use and abuse. These observations demonstrate both the need for and the complexity of analyzing the interactions of family and peer-group influences on the development of AOD abuse behaviors. Accordingly, future research into the etiology of alcoholism must examine more comprehensive, multifactorial models that integrate family environment, individual (i.e., biological and personality) risk factors, and peer group influences.

As they further elaborate these multifactorial models, scientists will likely identify significant correlations among some of the diverse risk factors. For example, personality characteristics such as behavioral disinhibition and sensation seeking appear to be risk factors for AOD abuse (Sher and Trull 1994). Because these personality dimensions can also affect the family environment, an interrelated cluster of genetic and environmental factors exists that influences a person’s trajectory toward alcohol problems. Behavioral-genetic research designs can clarify the independent and joint contributions of family environment and genetic influences to the development of undercontrolled behavior and, subsequently, behavioral disorders of childhood, adolescence, and young adulthood (McGue 1994).

Finally, carefully constructed intervention research will be extremely important in evaluating the validity of these developmental models and the malleability of personality and relationship dispositions associated with deviant outcomes. Nye and colleagues (1995) have conducted an early intervention study to reduce conduct problems of preschool-aged COA’s, hoping to thereby lower the risk for alcoholism among these children as they mature. Thus, this study represents a more “real-world” test of whether conduct problems truly mediate the influence of the family environment on the risk for alcoholism. In this context, researchers may do well to heed the words of a scientist who remarked: “If you want to understand something, try to change it.” (Bronfenbrenner 1977, p. 517).

## Footnotes

1 For additional background material and references, see Jacob and Leonard (1994) and Seilhamer and Jacob (1990).
